# Brain-derived neurotrophic factor (BDNF) in schizophrenia research: a quantitative review and future directions

**DOI:** 10.3934/Neuroscience.2023002

**Published:** 2023-03-17

**Authors:** Rozaziana Ahmad, Khairunnuur Fairuz Azman, Rosliza Yahaya, Nazlahshaniza Shafin, Norsuhana Omar, Asma Hayati Ahmad, Rahimah Zakaria, Adi Wijaya, Zahiruddin Othman

**Affiliations:** 1 School of Medical Sciences, Health Campus, Universiti Sains Malaysia, Kubang Kerian 16150, Malaysia; 2 Faculty of Medicine, Medical Campus, Universiti Sultan Zainal Abidin, Jalan Sultan Mahmud, 20400 Kuala Terengganu, Terengganu, Malaysia; 3 Department of Health Information Management, Universitas Indonesia Maju, Jakarta 12610, Indonesia

**Keywords:** brain-derived neurotrophic factor, schizophrenia, bibliometric analysis, Harzing's Publish or Perish, VOSviewer

## Abstract

This review aims to perform a bibliometric analysis of the research related to brain-derived neurotrophic factor (BDNF) in schizophrenia and offer suggestions for further work. Based on the keywords used, our study retrieved 335 documents for further analysis using a combination of three bibliometric techniques: co-word analysis, document co-citation analysis, and bibliographic coupling. A general rising trend in the number of publications was found in BDNF and schizophrenia research. Researchers from China and the United States have mostly researched BDNF and schizophrenia. Molecular Psychiatry is the most prestigious journal in the field of BDNF and schizophrenia research. The main topics and important research areas are cognition and the involvement of BDNF as a neurobiological marker (pathogenesis, therapy monitoring, and risk factors). Future research is anticipated to concentrate on relevant subjects, such as factors that affect BDNF levels or are connected to BDNF dysfunction in schizophrenia, as well as animal models of schizophrenia, in addition to cognition in schizophrenia.

## 1. Introduction

Schizophrenia is a complex psychiatric disorder with a prevalence rate of about 1 in 222 people (0.45%) among adults [1]. It affects people all over the world because of its long-term course, severe personality changes, and neurocognitive deficit such as working memory, attention and executive function that reduces the quality of life and causes a high percentage of patient disability [2],[3]. A neurocognitive deficit occurs in the early stage of schizophrenia and usually persists at all stages of the disorder [4]. It is recognised as a separate domain alongside positive and negative problems in several schizophrenia concepts, and it is an essential predictor of the disease's unfavourable prognosis and the development of persistent social dysfunction [5].

Multiple lines of evidence link altered neurotransmitter systems and brain connections with clinical observations of cognitive deficits and negative symptoms to disrupted neuroplasticity as the aetiology of schizophrenia [6]–[10]. One of the processes that are known to mediate neuroplasticity in the central nervous system is changes in neurotrophic factor activity [11],[12]. Neurotrophins such as brain-derived neurotrophic factor (BDNF), nerve growth factor (NGF) and glial cell line-derived neurotrophic factor (GDNF) are neuronal activity regulators involved in the development and maintenance of the mature nervous system, and as such, they may also contribute to the pathophysiology of schizophrenia [13].

Schizophrenia has become a rather wide discipline with its various research areas, which increases the number of studies every day and is one of the most researched topics in psychiatry. Earlier bibliometric studies mapped the author collaborations in schizophrenia [14] and the global scientific outputs of schizophrenia publications [15]. The former study involved 58,107 records from 2003 to 2012, downloaded from the Science Citation Index Expanded (SCI-Expanded) via the Web of Science. Based on their hierarchical clustering analysis, genetic research in schizophrenia was the main collaborative field. The latter study analysed 103,992 records also from the Web of Science database between 1975 and 2020. The authors demonstrated that some of the trend keywords that have been used in recent years include BDNF. BDNF is one of the most studied neurotrophins and is produced at the pre- and post-synaptic neurons [16]. It influences synaptic plasticity, causing significant changes in cognitive functioning, learning, and memory [17],[18]. It is also necessary for the development and maintenance of dopaminergic, GABAergic, cholinergic, and serotonergic neurons [19]. Variations in BDNF may cause changes in the brains of schizophrenia patients, such as a reduction in frontal grey matter volume and an increase in lateral ventricles and sulcal cerebrospinal fluid volume [20]. Additionally, BDNF has been investigated as a possible biomarker in cognition [21],[22] diagnosis and evaluation of schizophrenia [23]. To the best of our knowledge, this is the first article to carry out a bibliometric analysis of BDNF and schizophrenia publications. A few bibliometric analytic methodologies were used to address the following research issues.

1. What are the main subjects and themes of the research on BDNF and schizophrenia?
2. What are the important strong research areas in the field of BDNF and schizophrenia?
3. What are the research trends and changes in the authors' knowledge network related to recent literature on schizophrenia and BDNF?

## 2. Materials and methods

**Figure 1.**
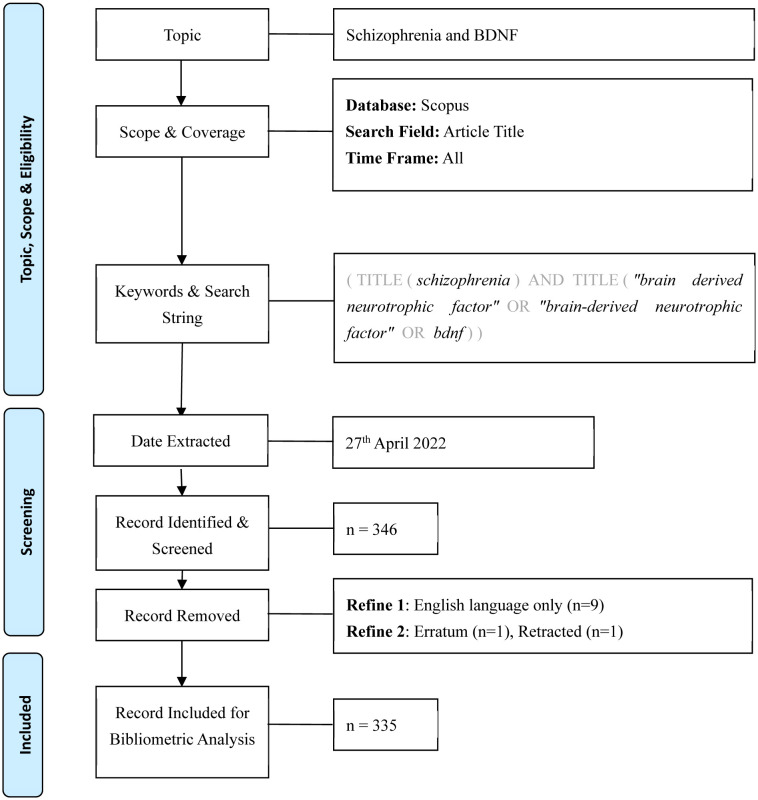
Flow diagram of the search strategy [26].

The data were retrieved on 27^th^ April 2022 from the Scopus database. A search of the relevant literature was carried out using the Scopus database because this database includes a larger number of publications and has more citations [24],[25]. We believed that there is sufficient information available to provide a sketch of the scientific landscape, research hotspots, and other pertinent details. The following search query in the article title was used: (TITLE(*schizophrenia*) AND TITLE (“*brain derived neurotrophic factor*” OR “*brain-derived neurotrophic factor*” OR *bdnf*)). Documents other than the English language (n = 9), retracted (n = 1) and the erratum (n = 13) documents were excluded (Figure 1).

A total of 335 documents were extracted from the Scopus database in Microsoft Excel (.xls), Research Information Systems (.ris) and Comma-Separated Values (.csv) format. The data in .ris and .csv format were analysed using Harzing's Publish or Perish [27] and VOSviewer version 1.6.17 [28] for descriptive and network analysis, respectively.

## 3. Results

### 3.1. Publication trends

**Figure 2.**
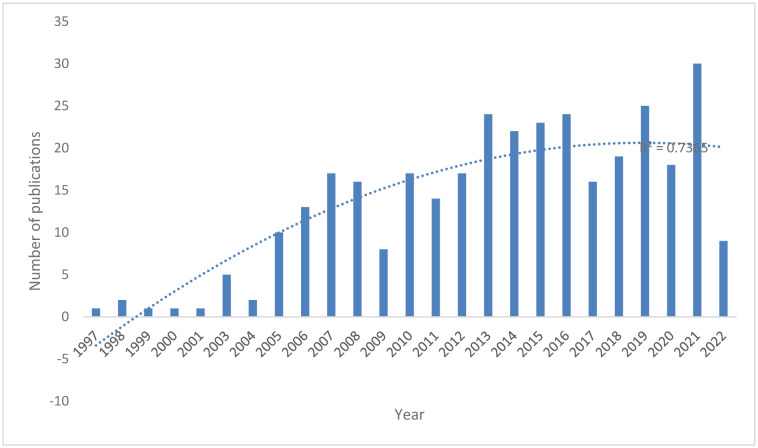
Trends in publications related to BDNF and schizophrenia.

Figure 2 depicts the annual trends in the number of publications. The publications barely reached two digits in 2005 and show an overall upward trend which peaks in 2021. The trend line shows that the number of publications increases polynomially (R^2^ = 0.7365).

### 3.2. Most productive authors

Most productive authors in that discipline are individual researchers who have made significant contributions to the growth and evolution of a research field. In BDNF and schizophrenia research, Zhang XY is ranked topmost with 22 publications and 938 citations, followed by Chen DC (14 publications, 736 citations), Tan YL (10 publications, 317 citations), Soares JC (10 publications, 277 citations), and Kosten TR (9 publications, 601citations) as shown in Table 1. The most productive authors are mostly from China and two from the USA and they co-authored some of the studies.

**Table 1.** Most productive authors.

Author's Name	Affiliation	Country	TP	NCP	TC	C/P	C/CP	*h*	*g*
Zhang, X.Y.	Chinese Academy of Sciences, Beijing, China	China	22	22	938	42.64	42.64	16	22
Chen, D.C.	Peking University, Beijing, China	China	14	14	736	52.57	52.57	13	14
Soares, J.C.	University of Texas Health Science Center at Houston, Houston, United States	USA	10	10	277	27.70	27.70	9	10
Tan, Y.L.	Peking University, Beijing, China	China	10	10	317	31.70	31.70	9	10
Kosten, T.R.	Baylor College of Medicine, Houston, United States	USA	9	9	601	66.78	66.78	8	9
Pillai, A.	VA Medical Center, Department of Research and Development, United States	USA	9	9	362	40.22	40.22	6	9
Xiu, M.H.	Peking University, Beijing, China	China	17	11	580	34.12	52.73	9	17
Huang, T.L.	Chang Gung Memorial Hospital, Genomic and Proteomic Core Laboratory, Taipei, Taiwan	Taiwan	8	8	217	27.13	27.13	6	8
Yoshimura, R.	University of Occupational and Environmental Health, Japan, Kitakyushu, Japan	Japan	8	7	110	13.75	15.71	6	8
Gama, C.S.	Universidade Federal do Rio Grande do Sul, Departamento de Psiquiatria e Medicina Legal, Porto Alegre, Brazil	Brazil	7	7	308	44.00	44.00	7	7
Hori, H.	Fukuoka University, Department of Psychiatry, Fukuoka, Japan	Japan	7	7	110	15.71	15.71	6	7
Nakamura, J.	University of Occupational and Environmental Health, Japan	Japan	7	7	163	23.29	23.29	6	7
Weickert, C.S.	University of New South Wales Faculty of Medicine, School of Psychiatry, Kensington, Australia	Australia	7	7	1191	170.14	170.14	6	7
Zhang, X.Y.	Institute of Psychology Chinese Academy of Sciences, Beijing, China	China	7	5	27	2.45	5.40	3	5

Source: created by the author based on Scopus and Harzing's Publish and Perish data

### 3.3. Most influential works

Table 2 listed the most cited publications on BDNF and schizophrenia in terms of the total number of citations. The journal Molecular Psychiatry published half of the papers listed in Table 2. Angelucci et al. [17] published in Molecular Psychiatry in 2005 was the topmost cited, with 445 citations. The second topmost cited article was an original article involving 57 post-mortem brains of patients with schizophrenia that was also published in Molecular Psychiatry in 2003 [29]. The third topmost cited article was a meta-analysis of 39 case-control studies encompassing psychiatric phenotypes: eating disorders, substance-related disorders, mood disorders, and schizophrenia, among others. This article was published in Biological Psychiatry in 2007 [30].

**Table 2.** Most cited articles.

No.	Authors	Title	Source	Year	TC	C/Y	References
1	Angelucci F, Brenè S, Mathé A	BDNF in schizophrenia, depression and corresponding animal models	Molecular Psychiatry	2005	445	26.18	[17]
2	Weickert CS, Hyde TM, Lipska BK, Herman MM, Weinberger DR, Kleinman JE	Reduced brain-derived neurotrophic factor in prefrontal cortex of patients with schizophrenia	Molecular Psychiatry	2003	435	22.89	[29]
3	Gratacòs M, González JR, Mercader JM, de Cid R, Urretavizcaya M, Estivill X	Brain-Derived Neurotrophic Factor Val66Met and Psychiatric Disorders: Meta-Analysis of Case-Control Studies Confirm Association to Substance-Related Disorders, Eating Disorders, and Schizophrenia	Biological Psychiatry	2007	342	22.80	[30]
4	Green MJ, Matheson SL, Shepherd A, Weickert CS, Carr VJ	Brain-derived neurotrophic factor levels in schizophrenia: A systematic review with meta-analysis	Molecular Psychiatry	2011	316	28.73	[31]
5	Hashimoto T, Bergen SE, Nguyen QL, Xu B, Monteggia LM, Pierri JN, Sun Z, Sampson AR, Lewis DA	Relationship of brain-derived neurotrophic factor and its receptor TrkB to altered inhibitory prefrontal circuitry in schizophrenia	Journal of Neuroscience	2005	316	18.59	[32]
6	Thompson Ray M, Weickert CS, Wyatt E, Webster MJ	Decreased BDNF, TrkB-TK+ and GAD67 mRNA expression in the hippocampus of individuals with schizophrenia and mood disorders	Journal of Psychiatry and Neuroscience	2011	248	22.55	[33]
7	Neves-Pereira M, Cheung JK, Pasdar A, Zhang F, Breen G, Yates P, Sinclair M, Crombie C, Walker N, St Clair DM	BDNF gene is a risk factor for schizophrenia in a Scottish population	Molecular Psychiatry	2005	241	14.18	[34]
8	Ho BC, Milev P, O'Leary DS, Librant A, Andreasen NC, Wassink TH	Cognitive and magnetic resonance imaging brain morphometric correlates of brain-derived neurotrophic factor Val66Met gene polymorphism in patients with schizophrenia and healthy volunteers	Archives of General Psychiatry	2006	224	14.00	[35]
9	Vinogradov S, Fisher M, Holland C, Shelly W, Wolkowitz O, Mellon SH	Is Serum Brain-Derived Neurotrophic Factor a Biomarker for Cognitive Enhancement in Schizophrenia?	Biological Psychiatry	2009	168	12.92	[36]
10	Krebs M, Guillin O, Bourdel MC, Schwartz JC, Olie JP, Poirier MF, Sokoloff P	Brain-derived neurotrophic factor (BDNF) gene variants association with age at onset and therapeutic response in schizophrenia	Molecular Psychiatry	2000	158	7.18	[37]

Source: created by the author based on Scopus and Harzing's Publish and Perish data

### 3.4. Keyword co-occurrence network

**Figure 3.**
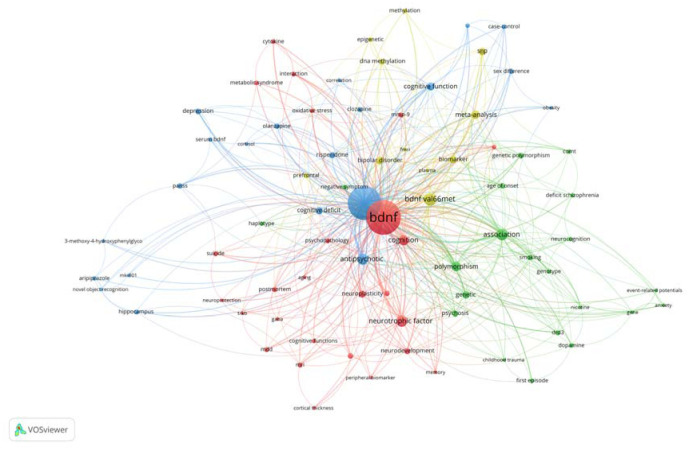
Co-occurrence network analysis. Data source: Scopus. Visualisation: VOSviewer.

In this study, VOSviewer was used to generate the keyword co-occurrence map. A total of 457 keywords were collected from our collection of 335 BDNF and schizophrenia publications. Only keywords that appeared at least three times were selected for keyword analysis. This criterion was met by 79 keywords out of 457. Our analysis indicates that the keyword network has four distinct clusters as shown in Figure 3: cluster one (red, 26 keywords), cluster two (green, 21 keywords), cluster three (blue, 21 keywords) and cluster four (yellow, 11 keywords).

### 3.5. Co-citation network of the most influential works

**Figure 4.**
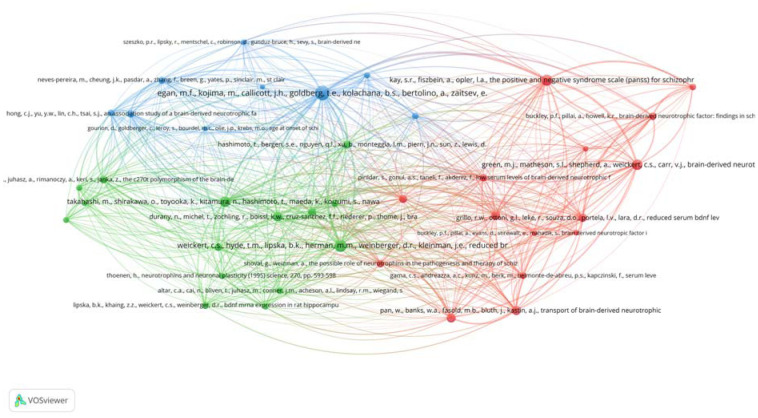
Co-citation network of a cited reference. Data source: Scopus. Visualisation: VOSviewer.

In this study, VOSviewer was used to generate the co-citation network map. The data was evaluated using the fractional counting approach with 10 as a minimum number of citations of a cited reference to create a co-citation network. The minimum cluster size was set at five. This criterion was met by 39 cited references out of 14144. The analysis resulted in a three-cluster network (Figure 4), the top 10 of which are listed in Table 3, while Table 4 summarizes BDNF in the schizophrenia co-citation network. The yellow cluster in the co-occurrence network analysis (Figure 3) correlated to the red cluster, which was associated with BDNF as a neurobiological marker in treatment monitoring (Figure 4). While the green and blue clusters exhibited a correlation with the blue and green clusters, respectively, these clusters were connected to the brain's BDNF distribution related to memory function and the function of BDNF polymorphism in the pathogenesis/risk of schizophrenia. The red cluster in the co-occurrence network analysis (Figure 3) was dispersed throughout all three clusters in Figure 4.

**Table 3.** Top 10 with the highest link strength, citations, and the number of links in the BDNF and schizophrenia field.

TLS	Citations	Links	Reference
68	77	37	[38]
45	51	37	[29]
38	40	36	[39]
37	46	31	[40]
32	32	37	[41]
31	42	30	[31]
30	30	38	[42]
29	31	29	[43]
27	29	34	[44]
24	26	31	[45]

TLS: total link strength

Source: created by the author based on the VOSviewer analysis

**Table 4.** Summary of BDNF and schizophrenia co-citation network (Clusters 1–3).

Cluster	Representative authors	Content	Core theoretical backgrounds
1 (Red, 15 articles)	Pirildar et al. 2004 [46]; Tan et al. 2005 [42]; Buckley et al. 2007 [47]; Gama et al. 2007 [48]; Grillo et al. 2007 [45]; Jindal et al. 2010 [49]; Buckley et al. 2011 [50]; Green et al. 2011 [31]	BDNF as a neurobiological marker	Treatment monitoring
2 (Green, 15 articles)	Thoenen 1995 [51]; Altar et al. 1997 [52]; Takahashi et al. 2000 [39]; Durany et al. 2001 [41]; Guillin et al. 2001 [53]; Lipska et al. 2001 [54]; Weickert, et al. 2003 [29]; Hashimoto et al. 2005 [32]	Distribution of BDNF in the brain	Role in memory
3 (Blue, 9 articles)	Egan et al. 2003 [38]; Hong et al. 2003 [20]; Neves-Pereira et al. 2005 [34]; Tan et al. 2005 [55]	Role of BDNF polymorphism	Pathogenesis/risk of schizophrenia

Source: created by the author based on the VOSviewer analysis

### 3.6. Bibliographical coupling network (document based)—2018–2022

**Figure 5.**
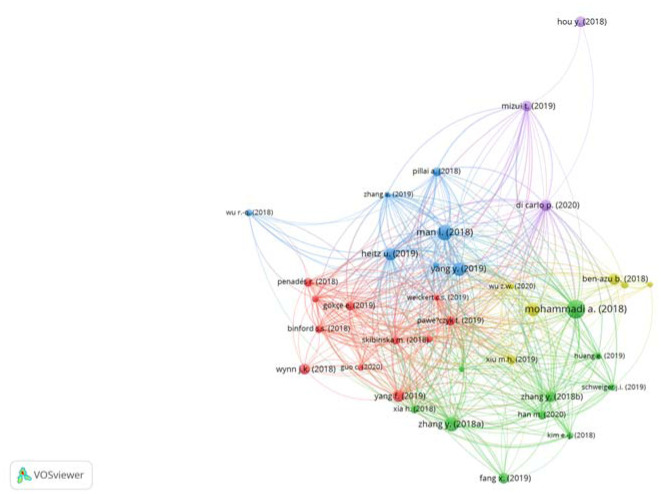
Bibliographic coupling network of the field by documents. Data source: Scopus. Visualisation: VOSviewer.

In this study, VOSviewer was used to generate the bibliographical coupling network map. The BDNF and schizophrenia publications from 2018 to 2022 were evaluated by bibliographic coupling using the full counting approach. A document with five as a minimum number of citations was selected to create a bibliographic coupling network. This criterion was met by 38 documents out of 345. Bibliographic coupling resulted in a five-cluster network (Figure 5), the details of which are listed in Table 5. The network's total link strength is 2570, meaning, the 37 document-based bibliographic networks appeared 2570 times jointly. There are a total of five clusters. The first cluster (red) shows the highest degree of coherence within the cluster while the fifth cluster (purple) shows the lowest degree of coherence within the cluster.

**Table 5.** Bibliographic coupling network of the field by documents.

Cluster	Pioneers	Title of work	Citations	TLS	Theme of the cluster
1 (11)	Yang et al. 2019 [56]	Sex difference in the association of body mass index and BDNF levels in Chinese patients with chronic schizophrenia	20	94	Factors affecting BDNF level or dysfunction
	Wynn et al. 2018 [57]	The effects of curcumin on brain-derived neurotrophic factor and cognition in schizophrenia: a randomized controlled study	17	15	
	Pawełczyk et al. 2019 [58]	An increase in plasma brain-derived neurotrophic factor levels is related to n-3 polyunsaturated fatty acid efficacy in first episode schizophrenia: secondary outcome analysis of the offer randomized clinical trial	13	91	
	Penadés et al. 2018 [59]	BDNF as a marker of response to cognitive remediation in patients with schizophrenia: a randomized and controlled trial	12	53	
	Gökçe et al. 2019 [60]	Effect of exercise on major depressive disorder and schizophrenia: a BDNF focused approach	11	85	
	Binford et al. 2018 [61]	Serum BDNF is positively associated with negative symptoms in older adults with schizophrenia	9	71	
	Skibinska et al. 2018 [62]	val66met functional polymorphism and serum protein level of brain-derived neurotrophic factor (BDNF) in acute episode of schizophrenia and depression	8	102	
	Atake et al. 2018 [63]	The impact of aging, psychotic symptoms, medication, and brain-derived neurotrophic factor on cognitive impairment in Japanese chronic schizophrenia patients	7	70	
	Faatehi et al. 2019 [64]	Early enriched environment prevents cognitive impairment in an animal model of schizophrenia induced by mk-801: role of hippocampal BDNF	7	24	
	Guo et al. 2020 [65]	ω-3pufas improve cognitive impairments through ser133 phosphorylation of creb upregulating BDNF/trkb signal in schizophrenia	6	28	
	Weickert et al. 2019 [66]	Increased plasma brain-derived neurotrophic factor (BDNF) levels in females with schizophrenia	5	104	

2 (10)	Mohammadi et al. 2018 [67]	Dysfunction in brain-derived neurotrophic factor signaling pathway and susceptibility to schizophrenia, Parkinson's and Alzheimer's diseases	49	88	BDNF dysfunction
	Zhang et al. 2018 [68]	Brain-derived neurotrophic factor as a biomarker for cognitive recovery in acute schizophrenia: 12-week results	30	85	
	Fang et al. 2019 [69]	Depressive symptoms in schizophrenia patients: a possible relationship between sirt1 and BDNF	17	24	
	Zhang et al. 2018 [70]	Interaction between BDNF and TNF-α genes in schizophrenia	16	78	
	Han et al. 2020 [71]	BDNF as a pharmacogenetic target for antipsychotic treatment of schizophrenia	12	87	
	Xia et al. 2018 [72]	Suicide attempt, clinical correlates, and BDNF val66met polymorphism in chronic patients with schizophrenia	8	86	
	Schweiger et al. 2019 [73]	Effects of BDNF val 66 met genotype and schizophrenia familial risk on a neural functional network for cognitive control in humans	7	55	
	Huang et al. 2019 [74]	BDNF val66met polymorphism and clinical response to antipsychotic treatment in schizophrenia and schizoaffective disorder patients: a meta-analysis	7	49	
	Kim et al. 2018 [75]	196g/a of the brain-derived neurotrophic factor gene polymorphisms predicts suicidal behavior in schizophrenia patients	7	48	
	Shoshina et al. 2021 [76]	Visual processing and BDNF levels in first-episode schizophrenia	5	63	

3 (7)	Man et al. 2018 [77]	Cognitive impairments and low BDNF serum levels in first-episode drug-naive patients with schizophrenia	34	163	BDNF as a neurobiological marker for cognition in schizophrenia
	Yang et al. 2019 [78]	Brain-derived neurotrophic factor is associated with cognitive impairments in first-episode and chronic schizophrenia	27	136	
	Heitz et al. 2019 [79]	Plasma and serum brain-derived neurotrophic factor (BDNF) levels and their association with neurocognition in at-risk mental state, first episode psychosis and chronic schizophrenia patients	22	149	
	Pillai et al. 2018 [80]	Predicting relapse in schizophrenia: is BDNF a plausible biological marker?	11	67	
	Wu et al. 2018 [81]	Effects of risperidone and paliperidone on brain-derived neurotrophic factor and n400 in first-episode schizophrenia	7	18	
	Nieto et al. 2021 [22]	BDNF as a biomarker of cognition in schizophrenia/psychosis: an updated review	5	99	
	Tang et al. 2019 [82]	Serum BDNF and GDNF in Chinese male patients with deficit schizophrenia and their relationships with neurocognitive dysfunction	5	94	

4 (6)	Wei et al. 2020 [83]	Interaction of oxidative stress and BDNF on executive dysfunction in patients with chronic schizophrenia	25	88	BDNF as a neurobiological marker for cognition in schizophrenia
	Ben-Azu et al. 2018 [84]	Involvement of gabaergic, BDNF and Nox-2 mechanisms in the prevention and reversal of ketamine-induced schizophrenia-like behavior by morin in mice	19	13	
	Xiu et al. 2019 [85]	Interaction of BDNF and cytokines in executive dysfunction in patients with chronic schizophrenia	13	48	
	Ahmed et al. 2018 [86]	Vinpocetine halts ketamine-induced schizophrenia-like deficits in rats: impact on BDNF and GSK-3β/β-catenin pathway	8	28	
	Wu et al. 2020 [87]	BDNF serum levels and cognitive improvement in drug-naive first episode patients with schizophrenia: a prospective 12-week longitudinal study	5	66	
	Xu et al. 2019 [88]	Applying vinpocetine to reverse synaptic ultrastructure by regulating BDNF-related psd-95 in alleviating schizophrenia-like deficits in rat	5	18	

5 (3)	Di Carlo et al. 2020 [89]	Brain-derived neurotrophic factor and schizophrenia	19	117	Non specific
	Mizui et al. 2019 [90]	Cerebrospinal fluid BDNF pro-peptide levels in major depressive disorder and schizophrenia	17	64	
	Hou et al. 2018 [91]	Schizophrenia-associated rs4702 g allele-specific downregulation of furin expression by mir-338-3p reduces BDNF production	17	2	

TLS: total link strength

Source: created by the author based on the VOSviewer analysis

## 4. Discussion

The BDNF and schizophrenia publications show an overall upward trend that peaks in 2021. The first article on the topic of BDNF in schizophrenia was published in the American Journal of Medical Genetics in 1997. This was the first scientific work indexed in the Scopus database to deal with the BDNF polymorphism as the possible candidate gene for schizophrenia [92]. This study was conducted on 60 unrelated Japanese schizophrenic patients and reported no evidence for the association between polymorphism and schizophrenia. Other studies included the Japanese population [93], the relationship between brain morphology and BDNF [94], SNPs within BDNF [95], and the effects of aripiprazole on BDNF [96]. The study also suggested further studies to be conducted on a larger number of subjects including other ethnicities. Case-control studies were later conducted on 130 French Caucasian schizophrenic patients and healthy volunteers [97] and 265 familial schizophrenic and healthy subjects in Irish families [98]. Both studies found no significant differences in allele frequencies or genotype distribution between patients and controls [97],[98]. Some of the later studies were also conducted in different populations such as Scottish [34], Dutch [99], Chinese [100]–[106], Asian [107], Russian [108], Egyptian [109],[110], Polish [111]–[113], Malay [114], and Turkish [115],[116]. These highlight the diversity in genetic research and the importance of studying BDNF and its related receptor genes in different populations.

The most prolific authors in a field can assist to identify scholars who have made significant contributions to the field's growth and progress. Most of the prolific authors are mostly from China and two are from the USA and they co-authored some of the studies. Their studies addressed a variety of themes which were related to BDNF and smoking [117]–[119], antipsychotic drugs [120],[121], symptoms of schizophrenia [121],[122], risk of schizophrenia [103], and cognition [22],[57],[79].

Most influential works are the most cited publications that have shaped the knowledge structure of a domain in that discipline. A review article by Angelucci et al. [17] published in Molecular Psychiatry in 2005 was the topmost cited, with a total of 445 citations. This review article described altered BDNF in schizophrenia, depression and animal models, as well as the effects of antipsychotic and antidepressive treatments on the expression of BDNF. The hypothesis was related to the malfunction of neurotrophic factors, which include BDNF, a mediator involved in neuronal survival and plasticity of dopaminergic, cholinergic, and serotonergic neurons in the central nervous system [17]. One of the animal models displaying structural brain deficits and supports the hypothesis that BDNF is implicated in the pathophysiology of schizophrenia was obtained by administering a single injection of methylazoxymethanol acetate (MAM) [123]. The second topmost cited article was an original article that was also published in Molecular Psychiatry in 2003. The study involved 57 post-mortem brains of patients with schizophrenia. They found a reduction in BDNF production and availability in the dorsolateral prefrontal cortex (DLPFC) of schizophrenics, and suggest that intrinsic cortical neurons, afferent neurons, and target neurons may receive less trophic support [29]. The third topmost cited article was published in Biological Psychiatry in 2007. The article was a meta-analysis of 39 case-control studies encompassing psychiatric phenotypes: eating disorders, substance-related disorders, mood disorders, and schizophrenia, among others. The study confirms the association of Val66Met with substance-related disorders, eating disorders, and schizophrenia [30]. The original and review articles about BDNF and its TrkB receptor [32],[33], BDNF polymorphism [124] and its role as cognitive markers [22],[36], and therapeutic response in patients with schizophrenia [37] make up the remaining top cited documents.

Keyword co-occurrence networks aid in the identification of relevant keywords used in publications within a knowledge domain and provide information on the domain's core research themes [125]. Our analysis resulted in four distinct clusters. The red cluster is the largest cluster with common keywords such as BDNF, neurotrophic factor, cognition, neuroplasticity, neurodevelopmental, and first-episode schizophrenia. These keywords indicate that the red cluster has publications that focus on the BDNF role related to the pathogenesis of schizophrenia. The research focused on the neurobiology of schizophrenia has emphasized the relevance of neurodevelopmental and neurotoxicity-related elements in the pathogenesis of this disease as reflected by the relevant keywords such as neuroplasticity, neurodevelopmental, and neuroprotection. Another important inference in this cluster is related to biomarkers for schizophrenia that include peripheral (BDNF/neurotrophic factor, matrix metallopeptidase 9 (MMP-9), cytokine, oxidative stress) and neuroimaging (magnetic resonance imaging (MRI), cortical thickness) biomarkers.

The green (second) and blue (third) clusters are the second largest cluster, each with 21 keywords. Association, polymorphism, genetic, psychosis, age of onset, genetic polymorphism and smoking are the common keywords in the green cluster. These keywords indicate that the green cluster has publications that focus on BDNF polymorphism associated with smoking/nicotine in schizophrenia as well as functional polymorphisms of other genes such as dopamine, drd3 and COMT. While the common keywords in the blue cluster include schizophrenia, antipsychotic, cognitive function, cognitive deficit, depression, risperidone, olanzapine, and positive and negative syndrome scale (PANSS). This cluster has publications that focus on the role of BDNF in monitoring the effects of antipsychotics on PANSS and cognition.

The yellow cluster is the smallest cluster and the main keywords are BDNF val66met, meta-analysis, bipolar disorders, biomarker, single nucleotide polymorphism (SNP), DNA methylation, prefrontal, and epigenetic. These keywords indicate that the yellow cluster has publications that focus on the role of BDNF polymorphism as a biomarker in the pathogenesis of schizophrenia and bipolar disorders. Another important inference from this analysis is that this cluster employed meta-analysis while studying the BDNF polymorphism as a biomarker.

Co-citation networks, which are commonly utilised in bibliometric network analysis, concentrate on the interactions or linkages between two publications [126]. When two papers are cited in a third paper, the former is said to as ‘co-cited.’ As more publications cite both of them, the co-citation relationship between them becomes stronger [127] and indicates strong study topics in a knowledge domain [128]. The first cluster (red) consists of 15 documents and six of which are listed as the top 10 with the highest total link strength (TLS) [31],[40],[42]–[45]. This cluster focuses on BDNF as a neurobiological marker and was used for diagnosis and treatment monitoring [31],[42],[45]–[50]. In terms of the time frame, this cluster has the widest range, comprises of documents published from 1987 to 2012.

Cluster 2 (green) has the same size as cluster 1 (red) and three documents are listed in the top 10 with the highest TLS [29],[39],[41]. This cluster consists of 15 documents that discuss the distribution of BDNF in the brain and its role in memory [29],[32],[39],[41],[51]–[54]. In terms of the publication date, this cluster includes works from 1991 to 2003. Finally, cluster 3 (blue) has the lowest number of documents and only one of the documents is listed in the top 10 with the highest TLS [38]. This cluster discusses mainly the role of BDNF polymorphism in the pathogenesis and risk of schizophrenia [20],[34],[38],[55]. It includes documents from 2001 to 2005.

Another widely used method for analysing and visualising knowledge networks in a topic is bibliographic coupling [129]. Two publications are bibliographically connected if they both quote the same third publication, i.e., they have the same reference list, with a higher commonality of publication suggesting a stronger coupling [129],[130]. It is a potential method of examining recent trends and changes in an author's knowledge network over time [128]. There are a total of five clusters. The first cluster shows the highest degree of coherence within the cluster. In the cluster (in red in Fig. 4), “Sex difference in the association of body mass index and BDNF levels in Chinese patients with chronic schizophrenia”, the most important article was the one by Yang et al. [56], which focused on the needs to consider sex when assessing the relationship between BDNF and metabolic syndromes in schizophrenia. Atake et al. [63] commented on the impact of ageing, psychotic symptoms, medication, and brain-derived neurotrophic factor on cognitive impairment in Japanese chronic schizophrenia patients. Both articles discussed the changes in BDNF levels due to different factors in two different ethics i.e. Chinese and Japanese schizophrenia patients. Weickert et al. [66] and Skibinska et al. [62] had the highest total link strength in researching the increased plasma BDNF in females with schizophrenia, and the association between val66met functional polymorphism and serum BDNF in schizophrenia and depression, respectively.

In the second cluster (in green in Fig. 4), the most important article was “Dysfunction in brain-derived neurotrophic factor signalling pathway and susceptibility to schizophrenia, Parkinson's and Alzheimer's diseases” by Mohammadi et al. [67]. The review paper highlighted the BDNF signalling pathway dysfunction in various brain diseases. The BDNF dysfunctions were also reported in schizophrenia and schizoaffective disorder by other studies in the same cluster [72]–[75].

In the third cluster (in blue in Fig. 4), “Cognitive impairments and low BDNF serum levels in first-episode drug-naive patients with schizophrenia”, was the most important article by Man et al. [77]. In both patients and healthy controls, no significant link was identified between BDNF and neuropsychological score. The study findings imply that excessive cognitive deficits are prevalent in the early stages of schizophrenia and low BDNF levels may have a role in the aetiology of schizophrenia, although not necessarily in cognitive problems. Other articles in the same cluster also reported the role of BDNF in the cognitive function of schizophrenia patients [68],[78],[79].

In the fourth cluster (in yellow in Fig. 4), three of the articles focused on the role of BDNF in the cognitive function of schizophrenia patients [80],[82],[84] as in the third cluster and had the highest total link strength. Two of the articles were related to ketamine-induced schizophrenia in animal models [84],[86]. While the fifth cluster (in purple in Fig. 4) is the smallest and exhibits the lowest degree of coherence within the cluster. The articles in this cluster had high total link strength except for the article by Hou et al. [91]. The article by Hou is related to the regulation of BDNF production by microRNAs (miRNAs).

## 5. Conclusions

Research on schizophrenia and BDNF has advanced significantly, particularly among experts connected to China and the USA. The main themes and strong research areas of BDNF in schizophrenia include its role as a neurobiological marker (pathogenesis, treatment monitoring, and risk factors), and cognition in schizophrenia. Recent years have seen an increase in interest in research on pertinent topics, such as factors that alter BDNF levels or are linked to BDNF dysfunction in schizophrenia, cognition in schizophrenia, and animal models of the disease. Future studies should look into the novel potential roles of BDNF in schizophrenia including regulation of inflammation, modulation of mitochondria function, epigenetic regulation, and regulation of neural oscillations.
